# Development of large-scale metabolite identification methods for metabolomics

**DOI:** 10.1186/1471-2105-15-S10-P36

**Published:** 2014-09-29

**Authors:** Joshua M Mitchell, Teresa W-M Fan, Andrew N Lane, Hunter NB Moseley

**Affiliations:** 1 Department of Molecular and Cellular Biochemistry, University of Kentucky, Lexington, KY 40356, USA; 2 Graduate Department of Toxicology, University of Kentucky, Lexington, KY 40356, USA; 3 Markey Cancer Center, University of Kentucky, Lexington, KY 40356, USA; 4 Resource Center for Stable Isotope Resolved Metabolomics, University of Kentucky, Lexington, KY 40356, USA

## Background

Large-scale identification of metabolites is key to elucidating and modeling metabolism at the systems level. Advances in metabolomics technologies, particularly ultra-high resolution mass spectrometry enable comprehensive and rapid analysis of metabolites, which is impractical to achieve by conventional methods. However, a significant barrier to meaningful data interpretation is the identification of a wide range of metabolites including unknowns and the determination of their role(s) in various metabolic networks. Our recent development of chemoselective (CS) probes to tag metabolite functional groups provides additional structural constraints for metabolite identification, but remains limited by the lack of functional group-resolved metabolite databases.

## Materials and methods

We have developed a novel algorithm to allow for the rapid detection of functional groups within existing metabolite databases such as KEGG Ligand and the Human Metabolome Database in order to create functional group resolved versions of both databases. These databases will allow for combined molecular formula and functional group (from CS tagging) queries to aid in metabolite identification based on accurate mass information without *a priori* knowledge.

## Results

An isomeric analysis of both HMDB and KEGG demonstrates a high percentage of isomeric molecular formulas, indicating the necessity of techniques such as CS-tagging with detection via MS and NMR to help assign specific metabolites and their isotopologue and isotopomer distributions based upon both molecular formula and distinct composition of functional groups. Furthermore, these two databases have only moderate overlap in molecular formulae. Thus, it is prudent to use multiple databases in metabolite assignment, since each of the major metabolite databases represents different portions of metabolism within the biosphere. *In silico* analysis of various CS-tagging strategies under different conditions for adduct formation demonstrate that the combination of FT-MS derived molecular formulas and CS-tagging can significantly increase the unique identification of isotopologues based on the entries in KEGG and HMDB databases.

